# Tregopathy in focus

**DOI:** 10.3389/fimmu.2025.1658140

**Published:** 2025-10-10

**Authors:** Vaishnavi Venkatachari Iyengar, Vijaya Gowri, Akshaya Sanjay Chougule, Prasad Taur, Manisha Rajan Madkaikar, Minnie Bodhanwala, Mukesh Manharlal Desai

**Affiliations:** ^1^ Department of Immunology, Bai Jerbai Wadia Hospital for Children, Mumbai, India; ^2^ Department of Immunology, Indian council of Medical Research (ICMR) - National Institute of Immunohematology, Mumbai, India

**Keywords:** Treg - regulatory T cell, autoimmune disease, polyautoimmunity, LRBA and CTLA-4 deficiencies, JAK- STAT signaling pathway, immune dysregulation, PIRD

## Abstract

Primary immune regulatory disorders are a newly coined term for a group of disorders in which autoimmune complications predominate. Herein, we present a case series of 26 patients with various regulatory T-cell (Treg) pathway defects who presented with multiple autoimmune complications. Twenty-six patients with pathogenic variants in T regulatory pathway genes were included, and their clinical data were evaluated. The median age at onset was 4.25 years, and the median delay in diagnosis was 2 years. The male-to-female ratio was 17:9. Thirteen children had LRBA deficiency, five had CTLA4 defect, two had IPEX, two had Cluster of differentiation 25 (CD25) defect, two had signal transducer and activator of transcription 3 (STAT3) Gain of function (GOF), and two had Fermitin family member 1 (FERMT1). Autoimmune cytopenia was the most common form of autoimmunity observed. Other autoimmune diseases included autoimmune hepatitis, inflammatory bowel disease, enteropathy, type 1 diabetes mellitus, thyroiditis, central nervous system (CNS) vasculitis, glomerulonephritis, and dermatitis. Most patients had evidence of lymphoproliferation with generalized lymphadenopathy and/or hepatosplenomegaly; 7/21 had hypogammaglobulinemia, 13/22 had low B-cell subsets, and 6/22 had low Cluster of differentiation 3 (CD3) levels. The treatments were diverse and included corticosteroids, cyclosporine, azathioprine, cyclosporine, and rituximab. After diagnosis, 12 patients were started on mTOR inhibitors, four on abatacept, and two on JAK inhibitors, with better control of autoimmunity. Five children underwent HSCT, and four are currently doing well. Patients with Treg deficiency present a broad range of clinical manifestations. A high index of suspicion for a monogenic cause of polyautoimmunity in early childhood can reduce delays in diagnosis. With the increasing availability of targeted therapies, the outcomes of these patients can be significantly improved.

## Introduction

Resistance to microbial pathogens is a key factor in evolutionary processes. Therefore, immunity gene clusters are hotspots of positive selection across species (1). The adaptive immune system not only responds to an enormous diversity of microbes but also provides optimal accommodation to the body’s commensals and fetus, while maintaining robust unresponsiveness to self-antigens. Thus, evolution has allocated significant assets to safeguard against self-reactivity, with the chief assets being T regulatory cells (Tregs). These mysterious thymic lymphocytes were characterized as carrying the cell surface receptor CD25 by Sakaguchi in 1995 (2), which led to the isolation of human Tregs in 2001 (3, 4), both in the thymus and peripheral blood of healthy individuals. The term tregopathies (5) was first introduced in 2018 and encompasses a group of inborn errors in immunity (IEI), in which the affected immune regulatory target is the Treg cell itself. In the latest 2024 IUIS classification, this list includes gene defects in forkhead box P3 (FOXP3) (X-linked), IL2RA (autosomal recessive [AR]), IL2RB (AR), cytotoxic T lymphocyte antigen 4 (CTLA4; autosomal dominant [AD]), lipopolysaccharide-responsive and beige-like anchor protein (LRBA [AR]), DEF6 (AR), NBEAL2 (AR), STAT3 GOF (AD), BACH2 (AD), FERMT1 (AR), and IKZF1 GOF (AD) (6). Herein, we present a case series of 26 patients with various Treg pathway defects who presented with multiple autoimmune complications and a review of the literature.

### Definitions

Clinically, patients were categorized as having autoimmune lymphoproliferative syndrome (ALPS)-like, common variable immunodeficiency (CVID)-like, and immune dysregulation, polyendocrinopathy, enteropathy, X-linked syndrome (IPEX)-like disorders. We defined ALPS-like Tregopathy as those having autoimmunity, especially autoimmune cytopenia (AIC), and lymphoproliferation (with negative ALPS evaluation). IPEX-like was defined as enteropathy and endocrinopathy, with or without other autoimmune manifestations. CVID-like was defined as chronic or recurrent infections with or without autoimmunity and lymphoproliferation (7). Lymphoproliferation was defined as the presence of at least two sites of hepatomegaly, splenomegaly, and/or lymphadenopathy.

### Statistical analysis

To describe the cohort, values are expressed as numbers, percentages, means, or medians, as appropriate.

## Methods

This was a retrospective analysis of 26 patients with a genetic diagnosis of Tregopathy. We reviewed the institutional medical records, including clinical findings, demographics, laboratory data, and treatment history. Diagnosis was confirmed by the causative genotype and additional segregation analysis or functional studies using flow cytometry, where available. Patients with variants of uncertain significance (VUS) in the Tregopathy gene without strong clinical correlation were excluded. This study was approved by the institutional ethics committee. Genetic testing was outsourced to a CAP/CLIA-accredited private laboratory. Variant calling and annotation were conducted using standard bioinformatics pipelines as per the laboratory protocol. Variant classification followed American College of Medical Genetics and Genomics (ACMG) Association for Molecular Pathology (AMP) 2015 guidelines (8), incorporating data from public databases (e.g., gnomAD, ClinVar) and *in silico* prediction tools (e.g., SIFT, PolyPhen-2, CADD). The final classification of variants as pathogenic or likely pathogenic was based on the laboratory’s interpretation following ACMG-AMP criteria. Extended family screening was performed for the identified variant in the index patient by Sanger sequencing to identify additional affected family members. Age-specific reference ranges were applied for all immunoglobulin levels, lymphocyte subsets, and Treg frequencies (9, 10). Flow cytometry was conducted using validated antibody panels, including CD3, CD4, CD8, CD19, CD16/56, CD25, and CD127, with appropriate gating strategies based on current clinical immunology guidelines (11).

## Results

**Figure 1:**
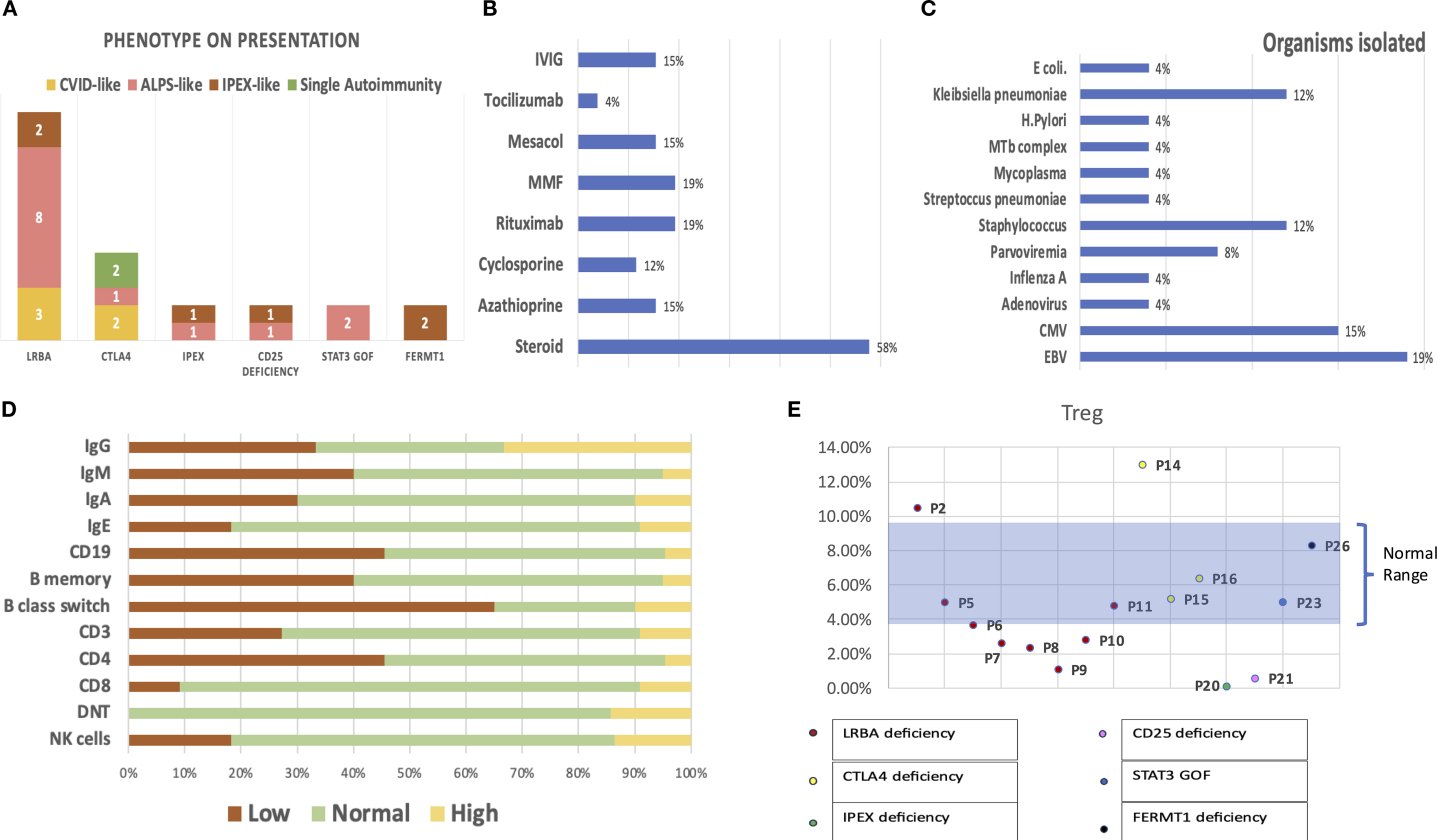
**(A)** Presentation of patients with each genetic defect in Tregopathy. **(B)** Polytherapy with multiple immunomodulatory agents prior to the diagnosis of Tregopathy. **(C)** Infectious susceptibility in the cohort: organisms isolated in the cohort and their relative proportions. **(D)** Immune evaluation: percentages reflecting the proportion of patients with abnormal values in each parameter. **(E)** Graph showing Treg estimation performed in 15/26 patients, with data labels denoting each patient.

Twenty-six patients were included in the study: 13 with LRBA deficiency, five with CTLA4 deficiency, and two each with IPEX, CD25, STAT3 GOF, and FERMT1 deficiency. The patient characteristics, variant details, therapies, and outcomes are summarized in **Table 1**. The median age at onset was 4 years and 3 months (range: 2 days to 40 years), with a median delay in diagnosis of 2 years (range: 1 month to 27 years). The median delay was greatest for patients presenting with a CVID phenotype (2 years; range: 2–27 years), followed by those with an ALPS-like phenotype (1.7 years; range: 0.5–8 years), and least for patients with an IPEX-like phenotype (10 months; range: 1.5 months to 2 years). There was a male preponderance (M:F = 1.8). Among the patients, 13/26 (50%) were born of consanguineous marriage, and 8/26 had a family history of autoimmunity or infection leading to death. **Figure 1A** shows the presentation of the patients with each genetic defect.

**Table 1:** Summary of patient characteristics, therapy, and outcome.

Patient	Diagnosis	Age at Onset/ Age at diagnosis/ delay in diagnosis	Clinical manifestation	Immunophenotype	Therapy details	Outcome
P1 (F)	LRBA c.7556_7557del (p.Pro2519ArgfsTer23) Homozygous	4.5 years / 5 years/ 0.5 years	Recurrent dermatitis, Lymphoproliferation (LP), Evans syndrome (ES), IBD	Normal immunoglobulin, normal number of T and B subsets. DNT 1.2% of T cells. Treg estimation not available (NA)	Steroid, Azathioprine, Cyclosporin, Sirolimus	Partial control (PC) with sirolimus, alive, refused HSCT
P2 (M)	LRBA c.4759_4762del ACTA (p.Thr1587ArgfsTer28) Homozygous	10 months/ 3years/ 2 years	Recurrent pneumonia (RTI), enteropathy, Type I diabetes (T1D), Hypothyroidism, ES, LP, hypogammaglobinemia	Low IgG and IgM, with high CD3, CD4, CD8 and NK counts. Low class switched B cells with DNT 5.67% of T cells. Treg (CD25+CD4+) : 10.5% of T cells. TTIgA>200, deamidated gliadin peptide(DGP):55.9(<20),Anti GAD antibody 201.25, Anti Islet positive	Steroids, azathioprine, Vincristin, rituximab, IVIG, insulin.	Refused HSCT, Died
P3 (M)	LRBA c.2449C>T (p.Gln817Ter) Homozygous	4 years /6 years /2 years	RTI, bronchiectasis, LP, ES, Hepatitis, hypogammaglobinemia	Panhypogammaglobinemia with low B cells, B memory and Class switched memory cells with normal T and NK subsets. Treg and DNT NA	Rituximab for EBV, IVIG, Sirolimus	Complete control (CC) with sirolimus, alive
P4 (F)	LRBA c.4759_4762delACTA (p.Thr1587ArgfsTer28) and c.4300_4301 del AT (p.Met1434ValfsTer33) Compound heterozygous in trans	9 months/ 15 months/ 6 months	RTI, ES, LP, Diarrhea with hypokalemia, hypocalcemia with tetany and hypomagnesemia, persistent hypoparathyroidism	Hypergammaglobinemia, low B cells, B memory and Class switched memory cells with normal T and NK subsets. DNT 1.9% of T cells, Treg NA	Steroids, Abatacept, Sirolimus, Hydroxychloroquine, IVIG	Alive PC with sirolimus (side effects: hypertriglyceridemia (TG) and recurrent loose stool and RTI), CC with abatacept. Successful haploidentical HSCT.
P5 (F)	LRBA c.3661-3662dup) (p.Thr1222LysFsTer4) Homozygous	9 years /22 years /13 years	RTI, IBD, extended ES, CNS vasculitis, arthritis, dermatitis, LP, glomerulonephritis, hypogammaglobinemia, ovarian teratoma.	Panhypogammaglobinemia with low B cells and B memory with normal T and NK subsets. DNT 1.84% of T cells and Treg 5%	Steroids, Cyclosporin, mycophenolate mofetil (MMF), poorly compliant on sirolimus	Alive, RTI with sirolimus
P6 (M)	LRBA c.4522C>T, (p.Gln1508Ter) Homozygous	2 months/ 2 years 8 months/ 2.5 years	RTI, ES, hypothyroidism, LP, multiple skin abscess	Hypergammaglobinemia, mildly reduced CD3 and NK cells. Normal B subsets. DNT 1.97%, Treg 3.7%. Anti TPO>1000, DCT+	Thyroxine, MMF	Died
P7 (M)	LRBA (c.2449C>T, p.Gn817Ter) Homozygous	10 years / 37 years/ 27 years	Hepatitis, enteropathy, LP, hypogammaglobinemia, Lipomas in upper limb and popliteal area	Panhypogammaglobinemia with low B cells. Normal T subsets and high NK cells. DNT 2.32% Treg 2.65%.	Refused treatment	Alive
P8 (M)	LRBA (c.2360 T>C, p.Leu787Pro) Homozygous	10 years/12 years/ 2 years	ES, recurrent otitis with moderate to severe hearing loss right and profound in left, LP, recurrent urticaria, 1 episode of cardiogenic shock, low IgA	Hypergammaglobinemia with low IgA levels. Low CD3, CD4, B cells and NK cells. 4.83% DNT. 2.4% Treg. DCT+	Steroids, MMF, sirolimus	Alive CC with sirolimus (mild HyperTG)
P9 (M)	LRBA (c.681C>A, p.Tyr227Ter Homozygous	5 months /20 months/ 15 months	Enteropathy and colitis, 1 episode of pneumonia	Normal immunophenotype except high B cells for age. 1.07% DNT, 1.1% Treg (low). TTGigA: 26.05 (<20)	Steroids, Mesalamine, poorly compliant on abatacept	Lost to follow up
P10 (M)	LRBA (c.4321 C>T, p.R1441W) Homozygous	6 years/ 8 years/ 2 years	RTI, low IgM, ES, hepatitis, IBD, LP, atopic dermatitis, onychomycosis of all nails	Low IgM and Low Ig E (0.31 IU/ml). Low CD4 cells, B memory and B class switched memory cells. DNT 1.47%, 2.8% Treg cells (low). DCT/ICT+, ASMA +	Steroids, Sirolimus, abatacept as a bridge to transplant	Alive PC with sirolimus, successful MUD HSCT
P11 (sister of P10)	LRBA (c.4321 C>T, p.R1441W) Homozygous	5 years/ 5 years/0	IBD, AIHA	Low CD4, naïve CD4 cells, B memory and B class switched memory cells. 1.1% DNT, Treg NA. DCT(0.5)/ICT(0.5+)	Noncompliant with sirolimus	Alive
P12 (F)	LRBA c.5779_5782del GACA, p.Asp1927fs*11 Homozygous	11 years/ 13 years/ 2 years	Pernicious anemia responded to B12, DCT positivity, RTI, ILD, diarrhea with hypokalemia, hypocalcemia with tingling and hypomagnesemia, low IgA and low IgG	Low IgG and IgA, Low CD3, CD4 cells, B cells, low class switched B memory cells. 1.78% DNT. DCT1+	Sirolimus, steroids, IVIG	Alive, CC with Sirolimus (s/e: oral ulcer)
P13 (F)	LRBA c.2573G>T, p.Arg858Met Homozygous	6 years/9 years/ 3 years	Severe Autoimmune thrombocytopenia (AITP) with two episodes of subdural hematoma, diarrhoea with hypocalcemia and tetany, hypokalemia, LP Low IgM and IgG	Low IgG and IgM. Low CD4 and CD4 naïve cells, B cells and NK cells. DNT 0.88%	Steroids, Sirolimus, rituximab, MMF	No control on sirolimus (s/e: oral ulcer), died
P14 (M)	CTLA4 c.346delA (p.Ile116SerfTer4) Heterozygous	10 years/ 12 years/ 2 years	ES, IBD, Hepatitis, LP, alopecia areata (**Figure 2B**)	Normal immunophenotype. DNT 2.6% of T cells (high), 13% Treg (high). DCT3+, ANA+	Mesalamine, steroids, Eltrombopag, oral budesonide, cyclosporine, rituximab, IVIG, azathioprine, Sirolimus, Abatacept	Alive PC with sirolimus (s/e HyperTg and RTI), CC with Abatacept, Successful HSCT
P15 (M)	CTLA4 c.401T>C p.M134T Heterozygous	2.5 years/ 3 years / 6 months	Extended ES, LP, hepatitis	Low CD4 and CD4 naïve cells. Normal DNT 1.92% and Treg 5.2%. anti SM +, DCt+, anti neutrophil antibody +	Steroid, Rituximab for autoimmunity and EBV viremia, Sirolimus	Alive CC with sirolimus
P16 (M)	CTLA4 c.221T>C Leu74Pro Heterozygous	26 years/ 36 years/ 10 years	Hodgkin lymphoma in a background of long standing lymphoproliferation, IBD, hypogammaglobinemia	Panhypogammaglobinemia, low CD3, CD4, CD8, B cells and B memory cells. Normal DNT 1.8% and Treg 6.4%	Chemotherapy for Hodgkin lymphoma	Alive
P17 (sister of P16)	CTLA4 c.221T>C Leu74Pro Heterozygous	40 years/42 years/ 2 years	IBD	No immune evaluation available	Noncompliant for sirolimus	Alive
P18 (Father of P15)	CTLA4 c.401T>C p.M134T Heterozygous	39 years/ 39 years/ 0	Hypothyroidism	Hypergammaglobinemia, no other immunophenotype available. AntiTPO+	Thyroxine, sirolimus	Alive (s/e HyperTg)
P19 (M)	FOXP3 intron 2 c.210+1G>A (5’ splice site) Hemizygous	2 years/3 years/1 year	AIHA, Nephritis, LP, dermatitis, multiple skin infections	No immune evaluation available. DCT/ICT +	Steroids, Azathioprine	Died 2 years post-transplant because of graft failure
P20 (M)	FOXP3 c.816+1G>T intron8 Hemizygous	3 months/ 1year 6 months/ 1 year 3 months	IBD/ enteropathy, Hypothyroidism, nephropathy, AIHA **Figure 2C**	Mild peripheral eosinophilia (range 100-1300 cells/cumm). High IgE (>2500IU/ml), low CD4 naïve low B cells. Normal DNT 1.86% and low Treg 0.1%. DCT+, ANA+	Steroids, mesalamine	Alive PC with sirolimus (s/e HyperTg, RTI, oral ulcer) refused HSCT
P21 (M)	IL2RA/CD25 c.131G>C (p.Gly44Ala) Homozygous	11 months/ 5 years 11 months/ 5 years	RTI, T1DM, dermatitis, EBV viremia	Persistent peripheral eosinophilia (593-5100 cells/cumm), Hypergammaglobinemia, low CD4 and Cd4 naïve cells, low CD8 and Cd8 naïve cells, low B memery cells, high NK cells. DNT 1.97% and low Treg 0.6%. AntiGAD 65+ 244.8, ANA+	Steroids, Sirolimus,IVIG	C with sirolimus (s/e RTI). Alive post MSD transplant
P22 (F)	IL2RA/CD25 exon6 c.668del p.Gln223fs Homozygous	1 month/ 8 months/ 7 months	Hypothyroidism, T1DM, enteropathy, dermatitis	Hypergammaglobinemia, no other immunophenotype available.	Insulin, Thyroxine	Died
P23 (M)	STAT3 c.2147C>T p.Thr716Met Heterozygous	6 years/ 6.5 years/ 6 months	Extended ES followed by polycythemia, short stature, LP (published 12)	Hypergammaglobinemia, low CD3, CD4, Bcells. Normal DNT 2.4%, Normal Treg 5%. DCT 3+. Elevated IL6 level elevated (432 pg/ml)	Steroid, tocilizumab, IVIG, Ruxolitinib, baricitinib	Alive CC with baricitinib
P24 (M)	STAT3 c.2240C>T p.Pro747Leu Heterozygous	4 years/ 11 years 9 months/ 7 years 9 months	Hypothyroidism, leucocytoclastic vasculitis, arthritis, LP, AIHA, short stature (published 13)	Low IgE (<0.1 IU/ml), high CD3 and CD8 cells. Normal DNT 1%. Anti Thyroglobulin Antibody- 248, DCT+. Normal IL 6 level	Steroid, MMF, Baricitinib	Alive CC with baricitinib
P25 (M)	FERMT1 c.1088delT (p.Leu363Trpfs*39) Homozygous	2 days/ 2 months/ 2 months	Persistent colitis/ IBD, multiple bullae on skin with denudation, pneumonia, E coli sepsis **Figure 2E** (left)	Low CD3, low Cd4, B cells, DNT 0.3%	Elemental formula	Died
P26 (F)	FERMT1 c.1264+1del Homozygous	1 months/ 2.5 months/ 1.5 months	Persistent colitis/ IBD, multiple bullae on skin with denudation, Klebsiella pneumoniae sepsis. **Figure 2E** (right)	Normal immunephenotype. 1.23% DNT and 8.3% of Treg	Elemental formula	Lost to follow up

A total of 15/26 (58%) had hematological autoimmunity (Evans syndrome [ES], *n* = 6; ES with autoimmune neutropenia [extended ES], *n* = 5; only autoimmune hemolytic anemia [AIHA], *n* = 5; only autoimmune thrombocytopenia [AITP], *n* = 1), 14/26 (54%) had gastrointestinal autoimmunity (inflammatory bowel disease [IBD], *n* = 10; enteropathy, *n* = 6; autoimmune hepatitis, *n* = 5), 8/26 (30.7%) had endocrinological autoimmunity (anti-TPO + hypothyroidism, *n* = 6; type 1 diabetes mellitus [T1DM], *n* = 3; short stature, *n* = 2; hypoparathyroidism [HypoPTH], *n* = 1), 3/26 (7%) had rheumatological autoimmunity (arthritis, *n* = 2; leucocytoclastic vasculitis [LCV], *n* = 1), 10/26 had dermatological manifestations (atopic dermatitis, *n* = 6; alopecia areata, *n* = 1; epidermolysis bullosa, *n* = 2), 3/26 had glomerulonephritis, 1/26 had interstitial lung disease (ILD) with lymphoid aggregates on lung biopsy, 1/26 had CNS vasculitis, and 18/26 had lymphoproliferation (LP). As a result of multiple autoimmune complications, these patients received polytherapy with multiple immunomodulatory agents, the details of which are shown in **Figure 1B**.

Of the 26 patients, 13 (50%) had either a life-threatening infection requiring admission or recurrent infections. The most common infections observed in the cohort were respiratory infections, especially in patients with LRBA deficiency, whereas patients with FERMT1 deficiency suffered gram-negative sepsis (*Escherichia coli*, *Klebsiella pneumoniae*). The isolated organisms are shown in **Figure 1C**.

Immune evaluations were performed on 22 children: 10/22 (45%) had CD19 lymphopenia, 6/22 (27%) had CD3 lymphopenia, 10/22 (45%) had CD4 lymphopenia, 2/22 (9%) had CD8 lymphopenia, 8/22 had a CD4/CD8 ratio < 1, 4/22 (18%) had NK lymphopenia, 7/21 had hypogammaglobulinemia, and 7/21 had hypergammaglobulinemia. **Figure 1D** shows an abnormality in the immune evaluation. Among the 15 patients, six had low Treg cells in the peripheral blood, including four of eight with LRBA deficiency, one of one with IPEX, and one of one with CD25 deficiency (**Figure 1E**). Detailed immunophenotypes and descriptions of autoantibodies for each patient are included in **Table 1**; **Supplementary Table S1**, and **Supplementary Figure S1**.

### Outcome

Targeted therapies were started in 16 patients: sirolimus in 12, abatacept in four, and a Janus kinase (JAK) inhibitor (ruxolitinib/baracitinib) in two. Two of these patients remained poorly compliant with targeted therapy to assess response. Of the evaluable patients, 57% (8/14) achieved complete control of autoimmunity, 35% (5/14) achieved partial control, and 7% (1/14) remained refractory to even targeted therapy. Sirolimus dosing ranged from 1.5 to 4.4 mg/m^2^/day, with a drug level range of 5.21–12.64 ng/mL to achieve a partial or complete response. Other details of the targeted therapies are listed in **Table 1**. Generally, these therapies were well tolerated. Among the patients, 40% had no side effects; respiratory tract infections (RTI) were observed in 33%, hypertriglyceridemia (TG) in 33%, oral ulcer in 13%, and loose stools in 6%. In total, 18/26 patients are alive, six have died, and two are lost to follow-up. Five underwent hematopoietic stem cell transplantation (HSCT), of whom one expired.

## Discussion

Tregs are specialized T cells that maintain peripheral tolerance and regulate immune responses by suppressing the functions and proliferation of various T-cell effectors. They suppress the production of proinflammatory cytokines and growth factors, as well as the expression of costimulatory molecules by themselves, expressing several T-cell coinhibitory surface molecules such as CTLA4, programmed cell death protein 1 (PD-1), and T-cell immunoreceptor with Ig and ITIM domains (TIGIT). In addition, Tregs regulate the activity of other cell types, such as B cells and monocytes (5).

Tregopathy is a monogenic disease that affects Treg homeostasis by altering critical signaling pathways, including those involved in survival, proliferation, differentiation, and activation (7). A brief overview of the different Tregopathies is presented in **Table 2** (14–31). For further in-depth understanding, readers are directed to several recent publications (5–7, 32, 33).

**Table 2:** Summary of published information on Tregopathy.

Serial number	Disease	Gene and inheritance	Clinical phenotype	Immune abnormality	Treatment	HSCT outcomes	Ref
1	IPEX	FOXP3 (X-linked)	Enteropathy, T1D, eczema, failure to thrive, cytopenia, thyroiditis, hepatitis	Absent or decreased FOXP3 expression in Tregs. Abnormal Treg numbers or function. Significantly elevated IgE. Elevated eosinophils	Sirolimus (response: 67%), calcineurin inhibitors, azathioprine, Mycophenolate mofetil (MMF), and methotrexate	Overall survival after HSCT: 73.2%	(14, 15)
2	CD25 deficiency	IL2RA (autosomal recessive [AR])	Enteropathy, T1D, dermatitis, recurrent respiratory infections (RI), autoimmune cytopenia, hepatitis, susceptibility to CMV	Low T, NK cells, poor T-cell proliferation, low CD25 expression	Sirolimus (used in 3 children and produced a response in all), IVIG, steroids, rituximab	Limited data (1 HSCT, failed engraftment followed by a second successful HSCT)	(16, 17)
3	CD122 (IL-2 receptor beta)	IL2RB (AR)	Cytopenia, enteropathy, vasculitis, RI, dermatitis, hepatitis	Low/Normal CD8. Low Tregs. Impaired T proliferation. Low CD122 cell surface expression	Rituximab. Sirolimus (used in 2 patients along with other agents, and both showed improvement)	Limited data (4 HSCT, 2 successful, 2 died)	(18, 19)
4	CTLA4 deficiency (ALPSV; CHAI)	CTLA4 (autosomal dominant, AD variable penetration and expressivity)	RI, inflammatory bowel disease (IBD), dermatitis, Granulomatous-lymphocytic interstitial lung disease (GLILD), lymphocytic infiltration of nonlymphoid organs, neurological affection, arthritis, Hemophagocytic lymphohistiocytosis (HLH), autoimmune cytopenia, endocrinopathy, enteropathy, Hodgkin’s lymphoma, non-Hodgkin’s lymphoma, gastric cancer.	CD3 lymphopenia. Low CD4 naïve. Low/normal Tregs. High circulating T follicular helper (cTFH), hypogammaglobulinemia, reduced to normal CTLA4 expression, and B lymphopenia. High B naïve, B cells, low B memory, high CD21low	Abatacept (93% responded fully), sirolimus (78% of GI manifestations and 62% of GLILD lesions resolved), IVIG, MMF, rituximab. HSCT.	Overall probability of survival at 3 years was 76.7%.	(20, 21)
5	LRBA deficiency (LATAIE)	LRBA (AR)	RI, IBD, skin lesions, GLILD, lymphocytic infiltration of nonlymphoid organs, neurological affection, arthritis, HLH, autoimmune cytopenia, endocrinopathy, enteropathy, Hodgkin’s lymphoma (HL), non-Hodgkin’s lymphoma	Hypogammaglobulinemia, low IgG and IgA defective class-switched B cells, expanded CD21 low B cells, CD3 lymphopenia, and low CD4 naïve. Low/normal Tregs. High cTFH. Low LRBA expression	Abatacept (CR in 74%), sirolimus (controlled cytopenia in 37.5% of patients, while ameliorating enteropathy in 57%), IVIG, MMF, rituximab. HSCT	64.2% showed complete remission	(20–22)
6	DEF6 deficiency	DEF6 (AR)	RI, IBD, cardiomyopathy, arthritis, EBV-associated lymphoproliferation, HL	Low CD4-naïve, normal or low Ig levels, CTLA4 cycling defective	Abatacept (1 patient responded completely)	No data	(23)
7	NBEAL2 deficiency	NBEAL2 (AR)	Grey platelet syndrome (macrothrombocytopenia, α-granule-deficient platelets, bleeding disorders), splenomegaly, and progression to myelofibrosis. Autoimmunity, EBV reactivation, HLH	Low CTLA4 expression in effector T cells alone	Abatacept (no data, conjecture by author [ref. 22] based on pathophysiology)	50% survival	(24–26)
8	STAT3 GOF (ADMIO, ADMIO1)	STAT3 (AD)	ILD, IBD, skin lesions, liver disease, arthritis, T1D, lymphoma, lymphoproliferation, infections, autoimmune cytopenias, and growth failure (frequently with enteropathy)	Increased double-negative T cells, decreased regulatory T cells, and increased numbers of TH17 cells, hypogammaglobulinemia, low IgE	Jak inhibitor (CR: 24.4%, PR in majority), IL-6 inhibitors	67% survival	(27, 28)
9	BACH2-related immunodeficiency and autoimmunity	BACH2 (AD)	RI, IBD, autoantibodies positive	Defective T-cell proliferation associated with a progressive T-cell lymphopenia, low FoxP3 expression level on Treg. Panhypogammaglobulinemia	Steroids, IVIG	No data	(29)
10	Kindler syndrome	FERMT1 (AR)	Skin blisters shortly after birth, followed by skin atrophy, pigmentation defects, photosensitivity, skin cancer, extensive mucosal involvement (hemorrhagic mucositis and gingivitis, periodontal disease, early tooth loss, and labial leukokeratosis), severe esophageal stenosis, anemia, and, rarely, infantile colitis	Intracellular accumulation of IgG, IgM, IgA, and C3 in colloid bodies under the basement membrane. Loss of αvβ6 integrin function leads to loss of TGF-β1 activation and failure to induce local intestinal Treg cells	Reducing trauma to the skin and use of moisturizers, limiting sun exposure, and intestinal manifestation may improve with age	No data	(30)
11	IKZF1 GOF	IKZF1 (AD)	Multiple autoimmunity T1D, enteritis, autoimmune hepatitis, Hashimoto thyroiditis, leukocytoclastic vasculitis, vitiligo, alopecia, and cytopenia. Bacterial infections, atopic and allergic diseases and lymphoproliferation	Normal B-cell numbers with normal to elevated serum immunoglobulin levels, including IgE. CD4 effector memory and CD8 TEMRA increased with increased Th2 differentiation, absence of effector Treg, and increased Tfh population. Eosinophilia	Lenalidomide, rituximab, sirolimus, and steroids, IVIG	1 patient cured with HSCT	(31)

### Lipopolysaccharide-responsive and beige-like anchor protein deficiency

LRBA deficiency was first described in 2012 (34). LRBA is a cytoplasmic protein that is ubiquitously expressed by almost all cell types but shows higher expression levels in immune effector cells. LRBA regulates intracellular vesicle trafficking and exocytosis of CTLA4 (35). It binds to the cytoplasmic tail of CTLA4 and maintains its intracellular stores, allowing rapid mobilization of the protein to the cell surface of Tregs (36, 37). CTLA4 is a critical and potent inhibitor of T-cell proliferation that serves as a “checkpoint” for immune responses. Accordingly, in the absence of LRBA/CTLA4 interaction, CTLA4-containing vesicles are shuttled to lysosomes for degradation (38). Our cohort consisted of 13 patients (P1–13, **Table 1**), 10 of whom were born by consanguineous marriage, with mutations spread across the *LRBA* gene. Six of these patients have been previously published as part of a larger cohort with limited clinical data (39). The median age at onset was 5 years (range: 2 months–11 years), and the median age at diagnosis was 6 years (range: 15 months–37 years), with a median diagnostic delay of 2 years. Our cohort had a later median age of onset but a much shorter diagnostic delay than recently published data on 212 patients with LRBA deficiency (21) (1.7 and 5 years, respectively). This could be attributed to two factors: a high degree of clinical suspicion owing to the results of a study we undertook (to screen all children, even with single autoimmunity, for monogenic causes; we found monogenic causes in 50% of these patients) (39) and the fact that a substantially larger proportion of our patients presented with ES (8/13, 61%). ES is associated with monogenic causes in approximately 60% of patients (40). As described in the literature (21), the most common autoimmunity in this cohort was AIC (77% *vs*. 70%), followed by autoimmune enteropathy or IBD (54% *vs*. 40%). The other autoimmune manifestations were endocrinopathy (T1D: 1, thyroiditis: 2, hypoparathyroidism: 1), alopecia/dermatitis (3/13), autoimmune hepatitis (3/13), arthritis (1/13), nephritis (1/13), ILD with lymphoid aggregates (1/13), and CNS vasculitis (1/13). However, nonmalignant lymphoproliferation was more commonly seen (76% *vs*. 54%) (21). Three children without overt features of enteropathy or IBD had recurrent episodes of diarrhea (infectious) associated with severe, long-lasting hypocalcemia, leading to tetany (2/3) and hypokalemia. Two patients were refractory to treatment and had an inadequate parathyroid response (low or normal PTH levels with hypocalcemia), which led to the suspicion of hypomagnesemia, as hypomagnesemia can blunt the parathyroid response (41). Both children had very low magnesium levels (0.7 and 0.8 mg/dL) and required parenteral correction, followed by normalization of potassium and calcium. Two case reports (42, 43) have described the same phenomenon of severe refractory hypokalemia following diarrhea in patients with LRBA, and we urge clinicians to consider magnesium levels, as they are not measured in most basic metabolic panels. All three patients improved with initial parenteral administration, followed by oral supplementation, although one of them had low PTH levels, suggestive of autoimmune hypoPTH.

Among the 13 patients, seven had recurrent pneumonia and three had recurrent otitis. The organisms isolated included gram-positive organisms, Epstein–Barr virus (EBV), and cytomegalovirus (CMV), which indicated a combined cellular defect or a side effect of multiple immunosuppressive agents, similar to other cohorts (44). Similar to the large cohort (21), 46% had at least two immunoglobulin isotype deficiencies. However, a larger percentage (69% in our cohort *vs*. 40% in other cohorts) (21) had either CD4/CD8/CD19/NK cell lymphopenia, which may be attributable to polytherapy with multiple immunosuppressive agents—a finding consistent with that seen in other cases in the cohort with CTLA4 defect, IPEX syndrome, and STAT3 GOF. Fifty percent (four of eight) had low Treg%, which is consistent with previous studies on LRBA deficiency (66% patients). Patients with LRBA deficiency not only have a numerical but also a functional defect in Treg cells (45). Sixteen percent had elevated double-negative T cells (DNT) (normal < 2.5% of T lymphocytes) *vs*. 22% in the literature, suggesting that children with LRBA deficiency can mimic ALPS and should be differentially diagnosed in cases with ALPS-like presentation.

**Figure 2:**
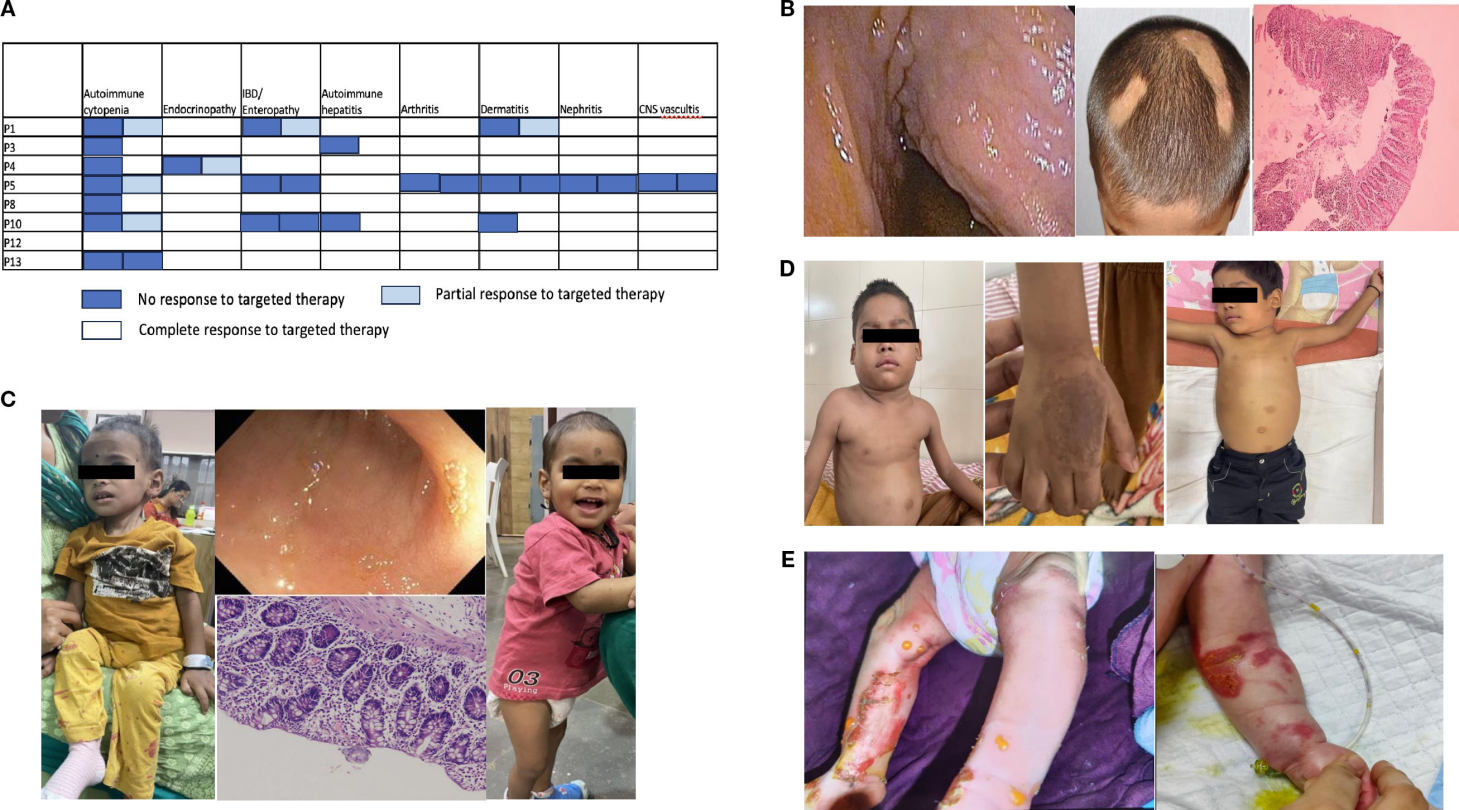
**(A)** Response to sirolimus with respect to patients and their autoimmunity. **(B)** Autoimmune manifestations in P14: colonoscopy image showing a cobblestone appearance, alopecia areata, and histopathology of colonic biopsy showing an increase in intraepithelial lymphocytes along with inflammation suggestive of colitis. **(C)** Clinical images of P20 before diagnosis showing extreme failure to thrive; terminal ileum showing flattening of villi; and colonic biopsy showing features of active colitis, followed by clinical improvement with steroid and sirolimus initiation. **(D)** Clinical images of P22 with cervical and axillary lymphadenopathy; hand images showing dermatitis; and postsirolimus regression of the lymph nodes. **(E)** Clinical images of P25 and P26 showing skin bullae followed by denudation and raw skin lesions.

Among the targeted therapies, sirolimus was administered to eight patients (one was poorly compliant). **Figure 2A** shows the response to sirolimus across different autoimmune manifestations. AIC and lymphoproliferation improved in all patients treated with sirolimus. As shown in previous studies (22), among different autoimmune diseases, AIC (62.5% of patients) responded best to sirolimus, followed by lymphoproliferation (57% of patients). Abatacept was administered to only three patients. In P9, poor compliance limited the assessment of response; P4 achieved complete disease control, and P10 received it as a bridge to transplant. Its limited use was primarily due to the high cost, which was beyond the reach of most of our patients. Sirolimus, on the other hand, is an inexpensive oral agent with readily available therapeutic drug monitoring; therefore, it is our first choice of targeted therapy for these patients.

### CTLA4 haploinsufficiency

Five patients (P14–18) in the cohort had mutations in exon 2 (a mutational hotspot) of the *CTLA4* gene encoding the extracellular domain. Two of these patients had been previously published as part of a larger cohort with limited clinical data (39). The youngest patient in our cohort presented at 2.5 years, whereas the median age of onset in the literature is approximately 10 years. This child (P15) presented with extended ES, lymphadenopathy, and EBV viremia. His father (P18) carried the same variant but was asymptomatic, later developing Hashimoto thyroiditis (anti-TPO > 100). Although younger patients have been reported (46), we hypothesized that EBV exposure triggered manifestations in the patient and his father. Other studies (47) have found that the seroprevalence of infectious agents did not differ between asymptomatic carriers and patients, but the authors did not explore whether age at exposure might play a role in the age of onset with the same underlying genetic susceptibility.

Sixty percent of patients had IBD/enteropathy, and 40% had AIC, which is similar to the proportion reported in the literature (21). Other autoimmunities observed were autoimmune hepatitis (2/5), alopecia areata (1/5), and Hashimoto’s thyroiditis. One patient had Epstein–Barr virus-encoded small RNAs (EBER+) Hodgkin lymphoma (HL). In a study by Egg et al. (48), HL was the most common malignancy associated with CTLA4 deficiency, and Epstein–Barr virus (EBV) triggers were identified in seven patients with lymphoma and three patients with gastric cancer. Thus, CTLA4 haploinsufficiency may uniquely predispose individuals to HL owing to abnormal immunosurveillance and an impaired ability to clear oncogenic viruses.

Sirolimus was administered to three patients, and two showed a complete response. P14 (**Figure 2B**) showed a partial response to sirolimus and was administered abatacept, which led to complete control, followed by successful HSCT. HSCT is regarded as the only long-term cure for CTLA4 insufficiency, with cure rates of 72%–76% (49, 50), but the treatment-related morbidity and availability of agents that can completely control the disease pose a dilemma regarding the time of referral. The ABACHAI trial (EudraCT No. 2019-000972-40; DRKS No. DRKS00017736) provides data on the long-term application and safety of abatacept. Although it was a single-arm, nonrandomized trial with a small sample size (*n* = 20) of mostly adults, it provides data showing that abatacept is a safe drug (five serious infections, none lasting > 3 months, and no EBV/CMV viremia-based events were reported), and that the CTLA4 haploinsufficiency (CHAI) morbidity score in most organ systems either remained stable or improved.

### Immune dysregulation, polyendocrinopathy, enteropathy, X-linked syndrome

The transcription factor FOXP3 is a master regulator of Treg cell development, differentiation, and immunosuppressive function; the expression level of the transcription factor FOXP3 is critical for maintaining immune homeostasis (51). Hemizygous mutations in FOXP3, which is located on the X chromosome, manifest in human male subjects as IPEX syndrome. The amount of DNA demethylation at FOXP3 promoter/enhancer regions is always higher in patients with IPEX syndrome. Therefore, even though FOXP3 expression level is normal in a few patients, Treg cells in these patients are unable to suppress T effector function (5). There were two children (P19, P20) with IPEX included in this cohort. One patient presented with AIHA and glomerulonephritis. He had been previously reported as part of a larger cohort with limited clinical data (39). Cytopenia and kidney involvement are uncommon manifestations of IPEX (14). The patient underwent HSCT, but subsequently had poor donor chimerism (10%) within 6 months, followed by death owing to graft failure. The other patient (**Figure 2C**) presented with enteropathy/IBD at 3.5 months of age, followed by diagnosis at 1.5 years. Pointers to clinical diagnosis were male sex, very early onset IBD, eosinophilia, and elevated IgE levels (5,406 IU/mL). During screening for additional autoimmunity, the patient was diagnosed with hypothyroidism and nephropathy with proteinuria. He was started on sirolimus at 2 mg/m^2^/day (9 ng/mL), which was reduced to 1 mg/m^2^/day (4 ng/mL) and later discontinued because of recurrent RTI and severe oral ulcer. He developed AIHA and was administered MMF with a poor response. Among the immunosuppressive agents, sirolimus is the only one known to control acute disease manifestations in 67% of patients (14). The patient’s parents refused HSCT. HSCT is the only available curative treatment, with an overall survival of 73%, but multiple studies (14, 52) found unexpectedly high graft failure (7%) or disease recurrence (33%), reducing the disease-free survival rate to 60%. Another study found similar remission rates in those with full and partial donor chimerism (53). Thus, strategies such as gene therapy with FOXP3-engineered Treg‐like cells (NCT05241444) may prove more effective than immunomodulatory agents or HSCT.

### CD25 deficiency

CD25 is a critical mediator of the interleukin-2 (IL-2) signaling pathway in Tregs. CD25 (i.e., IL-2 receptor α) binds with high affinity to IL-2, activating STAT5B-mediated signaling that eventually results in transcription of FOXP3. Defective IL-2 signaling thus severely affects the function of Treg cells, which is strictly dependent on FOXP3 (54). There were two children (P21,22) with CD25 deficiency in this cohort. The first patient was a girl with a history of sibling death from intractable diarrhea. She was found to have hypothyroidism during newborn screening. By 1 month of age, she developed watery diarrhea with severe ketoacidosis with low C-peptide (0.4 ng/mL) and was diagnosed with T1D. She died at 7 months of age from a bout of diarrhea and was posthumously diagnosed with CD25 deficiency. The second patient (**Figure 2D**) had recurrent episodes of RTI with wheezing starting at 9 months of age, generalized reactive lymphadenopathy since the age of 1.5 years, and dermatitis at 2 years of age. He was diagnosed with T1D at the age of 3.5 years and tested positive for anti-GAD65 antibody (244 nmol/L). He had poorly controlled diabetes (on a high dose of 2.2 units/kg/day of insulin) until he presented with a suspicious bilateral axillary and submandibular mass. On investigation, he was found to have direct Coombs test (DCT+) anemia (9 g/dL), hypergammaglobulinemia (2857 mg/dL), mild CD4 lymphopenia with severe reduction in naïve CD4 cells (11%), and very low Treg cells (0.6% of CD4 cells estimated by CD25^+^CD127 low cells, suggestive of near absence of CD25 expression) with normal DNT and benign reactive lymph nodes on histopathology. After diagnosis, the patient was started on intravenous immunoglobulin (IVIG) for recurrent RTI and sirolimus at 2 mg/m^2^/day. Similar to other patients with CD25 deficiency (16), surface expression was very low in our patient; hence, flow cytometric estimation of CD25 can be a rapid diagnostic test. The dose of sirolimus was increased from 2 to 3 mg/m^2^/day (10.8 ng/mL), but the patient developed pneumonia; hence, the dose was reduced back to 2 mg/m^2^/day with a drug level of 5.6–6.28 ng/mL. He showed a growth of 9.5 cm over 18 months, with reduced requirement for insulin (1 unit/kg/day) and achieved good glycemic control (HbA1c improved from 11.4% to 8.4%). Although data are limited to only three reported cases (17), sirolimus has demonstrated marked efficacy in children with CD25 deficiency, showing significant improvement in dermatitis and even reversal of diabetes when initiated early in the disease course. During illness, he also had intermittent EBV viremia that did not require any treatment, similar to other patients who had Herpesviridae susceptibilities (CMV, HHV-6) (16, 55, 56). The lymphoproliferation subsided completely (**Figure 2D**), with negative DCT results. He underwent successful HSCT with a matched sibling donor and is currently doing well with full donor chimerism 1 year posttransplant. Data on HSCT are limited in CD25 deficiency patients (16), and this is the second child to have undergone successful HSCT in the literature.

### STAT3 GOF

STAT3 activation upregulates the expression of secretion of cytokine signaling 3 (SOCS3), which inhibits STAT5, a positive regulator of FOXP3 and CD25 expression. STAT3 activation also inhibits tumor growth factor (TGF)-β-induced FOXP3 upregulation in naive CD4 T cells, polarizing them from Treg cells toward a potentially pathogenic TH17 phenotype. Both these mechanisms contribute to autoimmunity (5). There were two children (P23, P24) in this cohort. The first child presented with ES and LP but shortly thereafter developed polycythemia. This is the only patient reported in the literature with polycythemia. He had an elevated IL-6 level of 432 pg/mL and responded to the IL-6 inhibitor tocilizumab, followed by a JAK inhibitor. The second child presented with multiple autoimmunities that accrued over the years, including LP, LCV, arthritis, thyroiditis, and severe short stature. He also had very low IgE levels, which could serve as a marker for this disorder, as previously reported (57). Both children were successfully treated with JAK inhibitors. We encourage readers to check these articles for an in-depth description of both cases (12, 13). In a large cohort of patients with STAT3 GOF, most responded to JAK inhibitors (28). Patient survival post-HSCT is only 62% (27); hence, it remains unclear whether early intervention with HSCT is the optimal choice, especially for patients well controlled with oral JAK inhibitors.

### FERMT1 deficiency

Kindler syndrome is an exceptionally rare autosomal recessive genodermatosis, first identified in 1954 by Kindler (58). The FERMT1 gene product, kindlin-1, is a member of the kindlin protein family. Kindlin-1 is an intracellular protein that associates with αvβ6 integrin and serves as an intracellular activator of this receptor, leading to the release of active TGF-β1. αvβ6 integrin activation suppresses antigen-induced Th2 responses and inflammation by induction of Tregs (59, 60). Kindlin-1 is an epithelial-specific protein expressed in the skin, periodontal tissue, and colon (61). Its loss leads to a defect in Treg induction at barrier sites and results in epithelial barrier injury and dysfunction. There were two infants (P25, P26) with Kindler syndrome who were included in this cohort. Both children presented shortly after birth with large skin bullae, followed by denudation (**Figure 2E**) and bloody diarrhea. Colitis is a rare and severe manifestation of this disease (30). Null mutations in the *FERMT1* gene give rise to neonatal-onset skin atrophy and acute and fulminant intestinal epithelial dysfunction (62). Both children received multidisciplinary care with avoidance of skin trauma, emollients, and dressings, but new skin lesions occurred during the hospital course. Enteral feeding was withheld, followed by gradual stepping up of feeds, which led to the recurrence of bloody stool. Both children developed sepsis from enteric organisms (**Table 1**), probably owing to loss of barrier function and bacterial translocation. One child died, whereas the other recovered from sepsis and was discharged on an elemental diet with instructions to avoid skin trauma and to maintain photoprotection. However, the patient was lost to follow-up.

### How we approach patients with immune dysregulation for possible Tregopathy and treatment strategies

**Figure 3:**
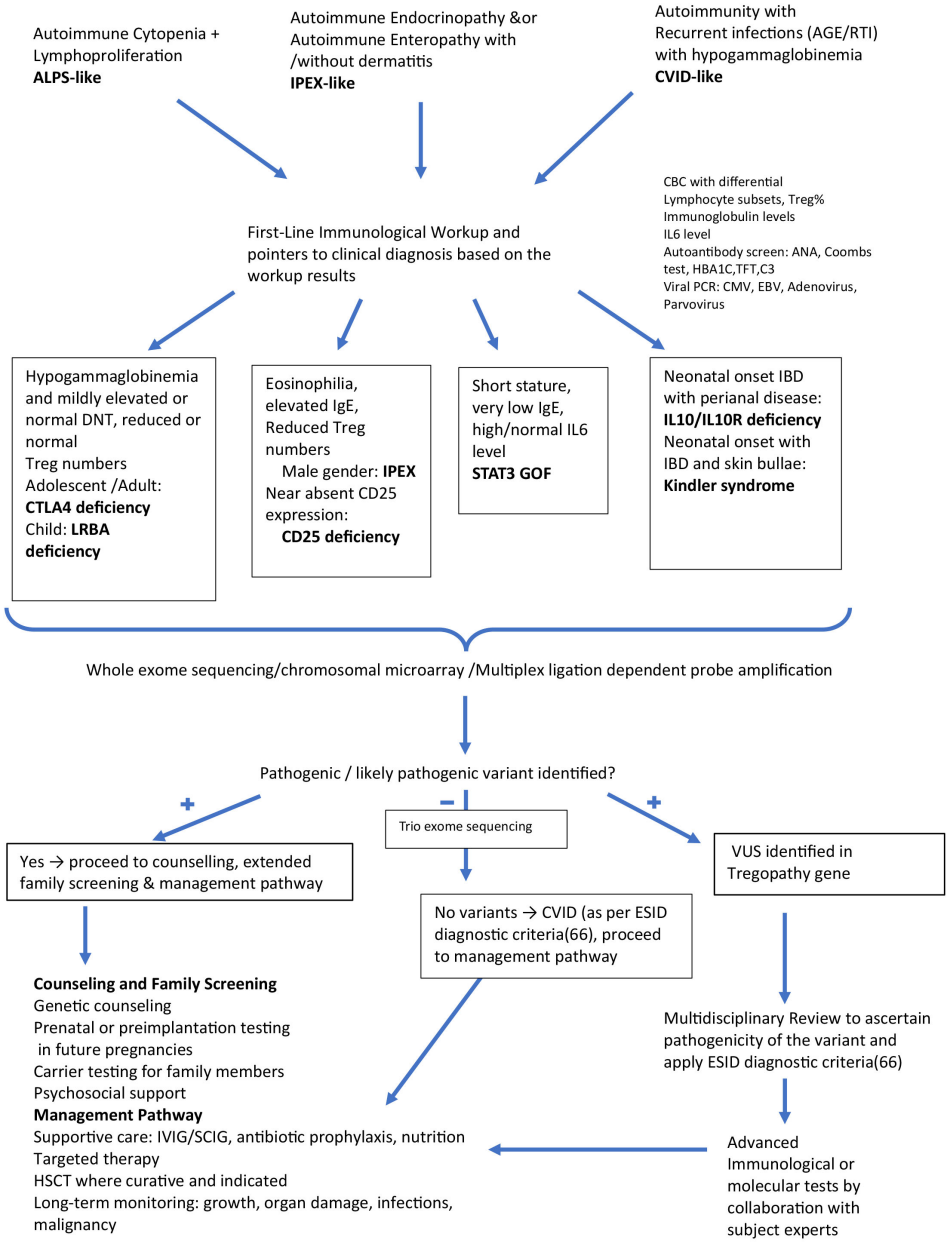
Algorithmic approach for evaluating patients with immune dysregulation: Identifying possible Tregopathies and outlining treatment strategies.

**Figure 3** delineates the approach we follow when investigating a suspected Tregopathy. All children or adults presenting with ALPS-like/CVID-like or IPEX-like phenotype undergo systematic evaluation to detect laboratory pointers indicative of an underlying Tregopathy: cytopenia, eosinophilia in complete blood count, abnormalities in CD3/CD4/CD8/NK/B cell and their subsets, Treg enumeration, immunoglobulin levels to identify hypergammaglobulinemia, especially high IgE and hypogammaglobulinemia. To identify undiagnosed autoimmunity, we perform screening autoantibody testing with antinuclear antibody (ANA) by immunofluorescence, direct and indirect Coombs test (DCT/ICT), C3 level, HbA1c, thyroid function test (TFT) in all patients suspected of having immune dysregulation. They are also screened for a few common viruses (adenovirus/CMV/EBV) to identify infection susceptibility and treat them before initiating targeted therapy. All patients undergo genetic testing depending on the presence or absence of syndromic features (if syndromic features are present, MLPA/chromosomal microarray is preferred; if not, whole-exome sequencing is undertaken). Due to financial constraints, trio sequencing is rarely performed but is a viable option, especially if no pathogenic (P)/likely pathogenic (LP) variants are identified in the initial whole-exome sequencing. If P/LP variants are identified, then the family undergoes genetic counseling and extended family screening with Sanger sequencing. Specific targeted therapy, along with supportive care, IVIG, and antibiotic prophylaxis, is initiated. HLA typing for the index patient, parents, and siblings (where available) is also performed.

The mTOR inhibitor sirolimus is our first choice for patients with Tregopathy, with the exception of STAT3GOF, for which a JAK inhibitor is preferred, and FERMT1 defects. We initiated sirolimus treatment after a pre-therapy work-up that consisted of a baseline immunological test along with a complete blood count (CBC), renal function test (RFT), liver function test (LFT), autoimmunity work-up (as indicated in the flowchart), and fasting triglyceride (TG) and cholesterol levels. The treatment was then initiated at a dose of 2 mg/m^2^/day with a target trough level of approximately 10 ng/mL, which usually achieves good control over 1–2 months of continuous therapy. Drug levels were monitored 7–10 days after initiation and after any change in dosage. CBC, LFT, and RFT with electrolytes, as well as fasting TG and cholesterol levels, were monitored monthly until dosage adjustment to achieve optimum trough levels and then every 2–3 months. Antibiotic prophylaxis with sirolimus was not routinely initiated unless indicated for primary defects. Although RTI was the most common side effect, patients with many Tregopathies are already susceptible to RTI; therefore, it is difficult to attribute the risk to sirolimus alone. In one study (63), antibiotic prophylaxis did not modify the risk of infection with sirolimus. Hypertriglyceridemia was also a common side effect in our cohort and did not require therapy discontinuation, similar to other case series (64). Our patients responded to niacinamide alone at 250–500 mg three times a day.

Among JAK inhibitors, baricitinib is the most accessible, and we initiated it at a dose of 2–4 mg/day in two divided doses based on the FDA recommendations for use as monotherapy in children aged > 2 years with COVID-19 (65). We screened for hepatitis B, hepatitis C, and HIV by serology; CMV and EBV by polymerase chain reaction (PCR); and tuberculosis. Trimethoprim/sulfamethoxazole, acyclovir, and fluconazole prophylaxis were also initiated. After starting baricitinib, CBC, liver function, renal function, TG, and blood glucose levels were monitored. In a study (65), the most commonly reported side effects were infections such as RTI and herpes zoster; other rare complications included anemia, Human polyomavirus 1 (BK) nephropathy, and thromboembolism. Both our patients experienced no adverse effects. While the control of autoimmunity, either partial or complete, is achieved especially in patients with LRBA/CTLA4 deficiency, IPEX syndrome, and CD25 deficiency, they were also referred to the transplant unit for donor search initiation and fund generation for transplant. For children with STAT3 GOF, none have been referred to the transplant team, as their conditions are well controlled and HSCT survival outcomes in this disorder remain suboptimal (62%), with most patients achieving satisfactory disease control on JAK inhibitors (27, 28).

All patients with immunodysregulation were monitored for worsening autoimmunity using six-monthly DCT/ICT, C3, HbA1C, thyroid function tests, and ANA by indirect immunofluorescence (IF). They were also monitored for CMV, EBV, and BK viruses in the blood by PCR every 6 months.

Our study has several important limitations. First, it is a single−center, retrospective case series, which introduces selection bias, as our center is a tertiary referral facility likely to receive more severe or atypical cases. Second, reliance on retrospective data contributes to missing information and heterogeneity in clinical assessment, particularly due to varying timelines for diagnostics, follow−up, and availability of functional assays, thereby limiting comparative interpretation of outcomes. Despite these limitations, the study provides valuable insights into the clinical spectrum of genetically confirmed Tregopathies with polyautoimmunity in a real-world setting. A significant strength is the comprehensive clinical evaluation available at our center. Our hospital hosts more than 20 pediatric subspecialties, allowing comprehensive in-house evaluation for endocrine, neurological, hematological, and nephrological manifestations. This substantially reduced reliance on external records and enabled consistent multisystem assessment in patients with suspected polyautoimmunity.

In conclusion, autoimmunity with lymphoproliferation, even if temporally separated, should prompt clinicians to consider Tregopathy. Early diagnosis helps prevent morbidity, allows extended family screening, and enables careful related donor selection, as these diseases can have incomplete penetrance. Apart from LRBA deficiency, patients with other disorders had very few infections, which probably led to a delay in the diagnosis of an immunological disorder. Routine immunological tests, including Treg enumeration, may not be sufficiently sensitive to rule out Tregopathy; therefore, exome sequencing is essential. Targeted drug therapy (sirolimus and baricitinib) can help control the disease until HSCT or gene therapy is available.

## Data Availability

The original contributions presented in the study are included in the article/**Supplementary Material**. Further inquiries can be directed to the corresponding authors.
