# Supplementary material for: Tregopathy in focus
Source: Front Immunol. 2025 Oct 10;16:1658140. doi: 10.3389/fimmu.2025.1658140 (PMC12549579; doi:10.3389/fimmu.2025.1658140)
Supplement: Supplementary file 1 [file Supplementaryfile1.docx]

Supplementary Table S1: Detailed Immunophenotype characteristics of patients (numbers in red are low as per age cut off, numbers in purple are high as per age cut off) ^9,10^

| Patient | Serum IgG (g/L) | Serum IgA (g/L) | Serum IgM (g/L) | Serum IgE (IU/mL) | CD3+ | CD3+4+ | CD3+8+ | CD19+ | Memory B | Class Switch | NK cell | DNT of T lymphocytes | Treg ( CD4+CD25+CD127-) |
| --- | --- | --- | --- | --- | --- | --- | --- | --- | --- | --- | --- | --- | --- |
| P1 | 7.85 | 0.536 | 0.92 | 20.9 | 2103 | 1023 | 853 | 341 | 9.50% | 5.20% | 313 | 1.20% | NA |
| P2 | 3.55 | <0.239 | <0.168 | 6.04 | **4520** | **2534** | **1507** | 90 | 8.90% | 4.10% | **890** | **5.67%** | 10.5% |
| P3 | 3.20 | <0.246 | <0.172 | 252 | 2474 | 1185 | 1115 | 488 | 7.50% | 3.50% | 418 | NA | NA |
| P4 | **14.9** | 0.797 | 1.19 | NA | 5163 | 3184 | 1721 | 2324 | 5.1% | 1.90% | 602 | 1.90% | NA |
| P5 | 3.32 | 0.37 | 0.44 | NA | 1728 | 1213 | 423 | 92 | 5.15% | 0.36% | 37 | 1.84% | 5% |
| P6 | **19.67** | 1.5 | 1.84 | 18.85 | 1767 | 1046 | 613 | 789 | 7.95% | 3.33% | 43.96 | 1.97% | 3.70% |
| P7 | 3.31 | 0.28 | 0.19 | NA | 1435 | 556 | 801 | 21 | 15.40% | 1.69% | **561** | 2.32% | 2.65% |
| P8 | **26.59** | 0.57 | **5.38** | NA | 916 | 570 | 289 | 95 | 6.70% | 3.70% | 27 | **4.83%** | 2.40% |
| P9 | 5.93 | NA | 0.63 | 25.9 | 4216 | 2570 | 1456 | **2597** | 8.48% | 4.40% | 334 | 1.07% | 1.10% |
| P10 | 7.28 | 0.55 | 0.40 | 0.315 | 1959 | 670 | 1120 | 317 | 6.64% | 0.59% | 276 | 1.47% | 2.80% |
| P11 | 6.92 | 1.15 | 0.69 | NA | 1549 | 718 | 583 | 250 | 11.8% low total number | 2.47% | 270 | 1.10% | NA |
| P12 | 6.38 | <0.25 | 0.67 | NA | 868 | 453 | 366 | 86 | 8.70% | 1.34% | 133 | 1.78% | NA |
| P13 | 6.06 | 0.6 | 0.31 | NA | 1845 | 730 | 1034 | 246 | 10.78% n | 3.73% | 26 | 0.88% | Na |
| P14 | 6.75 | 0.67 | NA | NA | 2243 | 871 | 1267 | 158 | NA | NA | 211 | 2.60% | 13% |
| P15 | NA | NA | NA | NA | 1315 | 484 | 566 | 792 | 24.20% | **12%** | 413 | 1.92% | 5.20% |
| P16 | 4.83 | 0.38 | 0.36 | 17.2 | 581 | 278 | 278 | 82 | 2.42% | 0.63% | 139 | 1.80% | 6.40% |
| P17 | NA | NA | NA | NA | NA | NA | NA | NA | NA | NA | NA | NA | NA |
| P18 | **20.7** | **3.28** | 2.96 | NA | NA | NA | NA | NA | NA | NA | NA | NA | NA |
| P19 | NA | NA | NA | NA | NA | NA | NA | NA | NA | NA | NA | NA | NA |
| P20 | 7.58 | 0.91 | 0.78 | **5,406** | 4488 | 1498 | 2536 | 160 | **29.70%** | **23%** | 914 | 1.86% | 0.10% |
| P21 | **19.02** | 1.43 | 0.96 | 45 | 901 | 410 | 371 | 349 | 11% | 6% | **1465** | 1.97% | 0.60% |
| P22 | **13.7** | 0.16 | 0.89 | NA | NA | NA | NA | NA | NA | NA | NA | NA | NA |
| P23 | **20.1** | **3.78** | 1.67 | 105 | 1258 | 446 | 685 | 175 | NA | NA | 111 | 2.40% | 5% |
| P24 | 9.93 | 2.4 | 0.42 | <0.1 | **3449** | 1310 | 1790 | 349 | 11.80% | 3.10% | 480 | 1% | NA |
| P25 | NA | NA | NA | NA | 1620 | 721 | 660 | 418 | 8.20% | 0.50% | 198 | 0.30% | NA |
| P26 | NA | NA | NA | NA | 3515 | 2693 | 706 | 1111.44 | 4.49% | 2.05% | 218.5 | 1.23% | 8.3% |
