# Supplementary material for: Tregopathy in focus
Source: Front Immunol. 2025 Oct 10;16:1658140. doi: 10.3389/fimmu.2025.1658140 (PMC12549579; doi:10.3389/fimmu.2025.1658140)

Supplementary Figure S1: **Distribution of patient immunological parameters.**
Violin plots depict the distribution of serum immunoglobulins (IgG, IgA, IgM, IgE) and lymphocyte subsets (CD3⁺, CD3⁺4⁺, CD3⁺8⁺, CD19⁺, Memory B cells, Class Switch B cells, NK cells, DNT, and Treg) in patients. **Individual data points** are shown as dots. The **black diamond** indicates the **mean** for each marker, and the **error bars** represent the **standard deviation (SD)**. Facets are scaled independently for each marker.


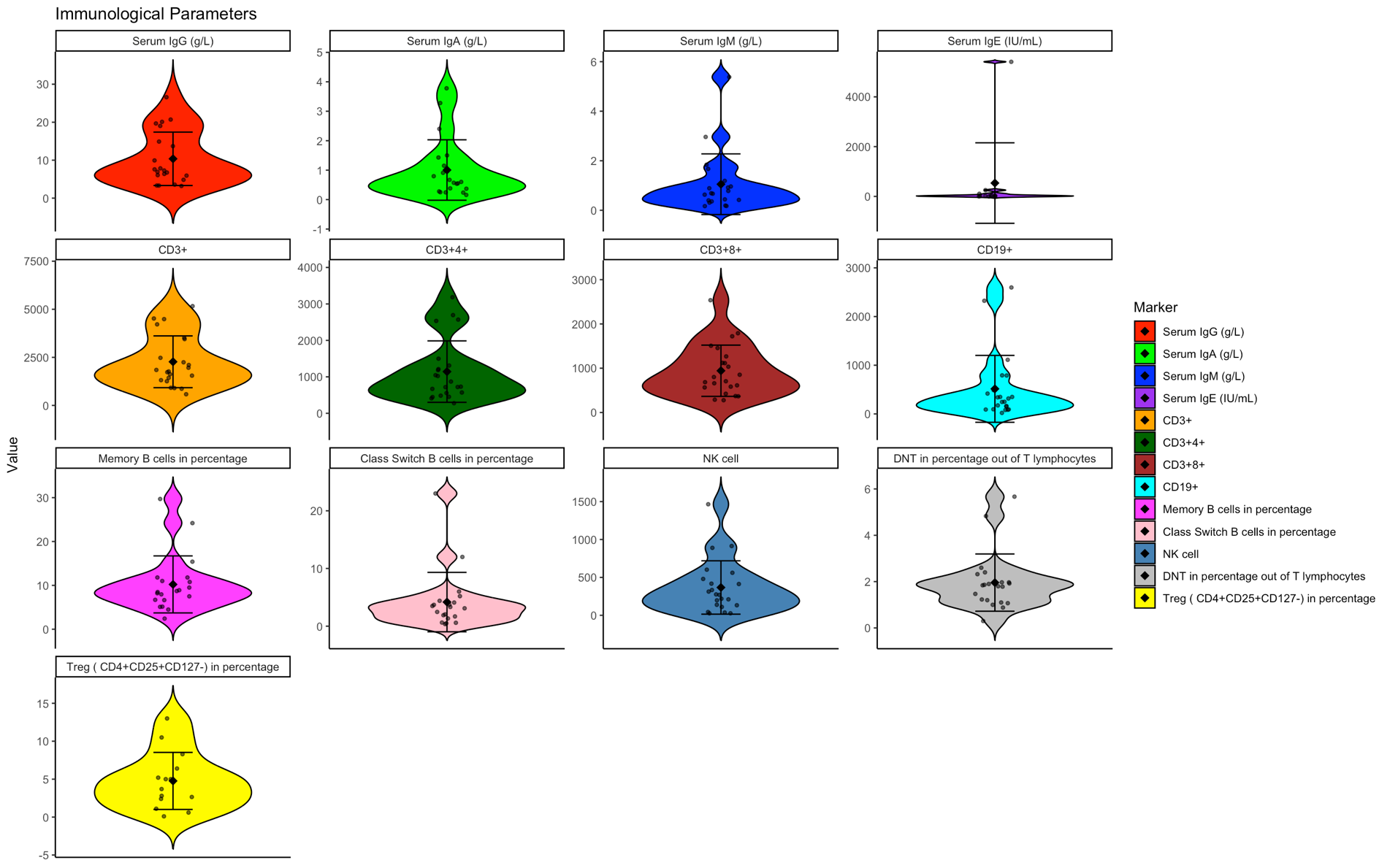

Supplement: Supplementary file 2 [file Supplementaryfile2.docx]
